# The effect of all-cause hospitalization on cognitive decline in older adults: a longitudinal study using databases of the National Health Insurance Service and the memory clinics of a self-run hospital

**DOI:** 10.1186/s12877-022-03701-4

**Published:** 2023-02-01

**Authors:** Dougho Park, Hyoung Seop Kim, Jong Hun Kim

**Affiliations:** 1 Department of Rehabilitation Medicine, Pohang Stroke and Spine Hospital, Pohang, South Korea; 2 Department of Medical Science and Engineering, School of Convergence Science and Technology, Pohang University of Science and Technology, Pohang, South Korea; 3 Department of Physical Medicine and Rehabilitation, National Health Insurance Service Ilsan Hospital, 100 Ilsan-ro, Goyang, 10444 Republic of Korea; 4 Department of Neurology, National Health Insurance Service Ilsan Hospital, 100 Ilsan-ro, Goyang, 10444 Republic of Korea

**Keywords:** Cognitive dysfunction, Hospitalization, Aged, National health programs

## Abstract

**Background:**

Cognitive decline is common in older adults and imposes a burden on public health. Especially for older adults, hospitalization can be related to decreased physical fitness. This study aimed to investigate the quantitative association between hospitalization and cognitive decline.

**Methods:**

This was a retrospective cohort study. We performed a longitudinal study by using the combined database from the Korean National Health Insurance Service (NHIS) and memory clinic data of its self-run hospital. We identified whether hospitalized, the number of hospitalizations, and the total hospitalization days through the claim information from the NHIS database. We also identified whether hospitalization was accompanied by delirium or surgery with general anesthesia for subgroup analysis. Primary outcome was the clinical dementia rating-sum of boxes (CDR-SB) score. Secondary outcomes were mini-mental state examination (MMSE) score, clinical dementia rating (CDR) grade, and Korean-instrumental activities of daily living (KIADL) score. Multivariable mixed models were established.

**Results:**

Of the 1810 participants, 1200 experienced hospitalization at least once during the observation period. The increase in CDR-SB was significantly greater in the hospitalized group (*β* = 1.5083, *P* < .001). The same results were seen in the total number of hospitalizations (*β* = 0.0208, *P* < .001) or the total hospitalization days (*β* = 0.0022, *P* < .001) increased. In the group that experienced hospitalization, cognitive decline was also significant in terms of CDR grade (*β* = 0.1773, *P* < .001), MMSE score (*β* = − 1.2327, *P* < .001), and KIADL score (*β* = 0.2983, *P* < .001). Although delirium (*β* = 0.2983, *P* < .001) and nonsurgical hospitalization (*β* = 0.2983, *P* < .001) were associated with faster cognitive decline, hospitalization without delirium and with surgery were also related to faster cognitive decline than in the no hospitalization group.

**Conclusion:**

Cognitive decline was quantitatively related to all-cause hospitalization in older adults. Moreover, hospitalizations without delirium and surgery were also related to cognitive decline. It is vital to prevent various conditions that need hospitalization to avoid and manage cognitive dysfunction.

**Supplementary Information:**

The online version contains supplementary material available at 10.1186/s12877-022-03701-4.

## Background

Cognitive impairment is common in older adults, and the prevalence of mild cognitive impairment and dementia is high in this group [1]. As the numbers of older people increase, the need for effective management and intervention for this population has been emerging [2, 3]. In particular, cognitive decline in older adults can affect their medical condition and functional level [4], which inevitably increases the demand for nursing care and facility usage [5, 6]. As a result, cognitive impairment is considered a critical public health issue, imposing a social burden through increased costs [7].

Researchers have focused on the relationship between hospitalization and cognitive decline in older adults [8]. In general, previous studies have shown that hospitalization is associated with faster cognitive decline in older adults [9, 10]. Concomitantly, the relationship between physical frailty and cognitive decline in older adults has also been studied. Yoon et al. [11] reported that physical function, muscle strength, and biomechanical measurements were associated with cognitive decline in a study of rural community-dwelling older adults. A cross-sectional study from Brazil also demonstrated that frailty phenotypes such as hand-grip strength and gait speed are associated with cognitive impairment in people aged ≥ 60 years [12].

South Korea operates a national health insurance service (NHIS) system, and all citizens are obliged to join the NHIS [13]. The NHIS database contains information claimed by medical service providers from the health insurance review and assessment service. The database includes information on hospital visits with primary diagnostic codes [14]. Furthermore, the Korean NHIS operates its own self-run hospital, and we can combine the hospital’s electrical medical records (EMR) with the NHIS database. This process is reviewed and controlled by the data linkage committee of the NHIS [15].

In this study, we assumed that not only hospitalization quantitatively related to cognitive dysfunction, but also hospitalizations can indirectly reflect the physical fitness state of older adults. To confirm these hypotheses, this study used data from the NHIS database combined with the EMR from its self-run hospital. Using the advantages of the database, we quantified hospitalization information and tried objectively to identify the relationship between cognitive decline and hospitalization. In addition, in terms of other contributing factors, we identified delirium and surgery during the hospitalization and investigated whether hospitalization with delirium and surgical treatment adversely affected cognitive decline.

## Methods

### Data source and participants

**Fig. 1:**
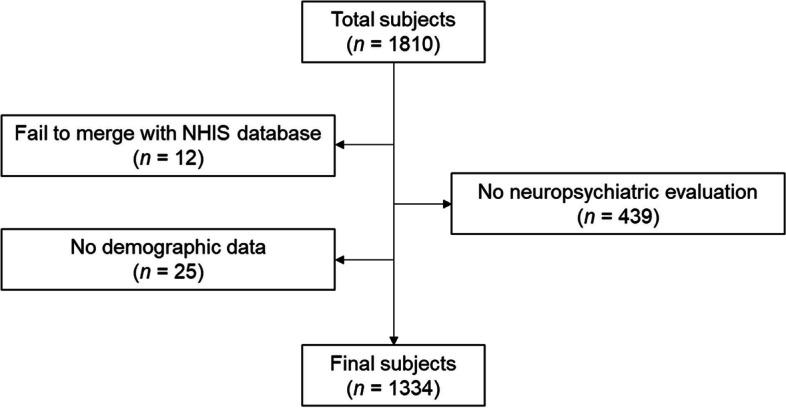
Flow chart of subject inclusion. Abbreviation: NHIS, National Health Insurance Service

This is a retrospective cohort study. The definition of the initial cohort was patients who 1) visited the memory clinic of NHIS Ilsan hospital and 2) completed apolipoprotein E (*APOE*) genotype screening; a total of 1810 subjects were extracted from January 2002 to December 2019. Then, we conducted a longitudinal study by combining the individual subject’s EMR from the hospital with the NHIS database. Cases not linked to the NHIS database (*n* = 12), cases in which neuropsychiatric evaluations were not completed (*n* = 439), and cases with missing demographic data (*n* = 25) were excluded from the final cohort. Finally, a total of 1334 subjects were included (Fig. 1).

This study was reviewed and approved by the Institutional Review Board of NHIS Ilsan Hospital (NHIMC 2022-06-014). Since the retrospective nature of this study with an anonymized database, the need for written informed consent was waived.

### Covariates and outcome

The primary outcome was set as the clinical dementia rating-sum of boxes (CDR-SB) score on the subsequent follow-up evaluation from the time of cohort entry. The secondary outcomes were set as the subsequent follow-up evaluations such as mini-mental state examination (MMSE) score, clinical dementia rating (CDR) grade, and Korean-instrumental activities of daily living (KIADL) score from cohort entry. Experienced neuropsychologists performed all neuropsychiatric evaluations.

Hospitalization-related information was the variable of interest. We identified whether hospitalized, the number of hospitalizations, and total hospitalization days from the claim data of the health insurance review and assessment service. The primary diagnosis of each hospitalization was obtained, and we categorized them according to the international classification of disease-10 clinical modification (ICD-10-CM) [16]. We excluded hospitalization data that were claimed from long-term care hospitals (convalescent or mental hospitals).

All participants underwent screening for *APOE*ε4 and *APOE*ε2 genotypes. We identified hypertension and diabetes as vascular risk factors, and the subcortical ischemic burden was further identified according to the criteria of Erkinjuntti et al. [17].

We confirmed whether hospitalization was accompanied by delirium or surgery with general anesthesia for subgroup analysis. We identified hospitalization with delirium as a claim code for antipsychotics prescription after hospitalization to persons who had not been prescribed them before. Surgery was identified by the related acting codes claimed within the hospitalization period.

### Statistical analysis

We expressed continuous variables as mean ± standard deviation and used an independent *t*-test to compare between groups. Categorical variables were expressed as frequency and proportion, and the chi-squared test was used for the comparison analysis.

We established mixed models with a random intercept and random slope, using covariates as fixed effects. In the models, we evaluated the interaction between hospitalization experiences and times to check cognitive decline differences according to those events. The compound symmetric was used as the covariate matrix. Three different incremental mixed models were constructed to check effects of covariate adjustments. Model I included sex and baseline CDR-SB score as covariates. Model II added age and presence of hypertension and diabetes as additional covariates to Model I. In Model III, we introduced education years and severities of vascular burdens as additional covariates to Model II.

A *P* value < 0.05 was considered statistically significant. We performed a complete case analysis to deal with missing values. The statistical analyses were performed using SAS enterprise guide (version 7.15; SAS Institute Inc., Cary, NC, USA) and R software version 4.0.3 (R core team, R Foundation for Statistical Computing, Vienna, Austria).

## Results

### Baseline characteristics

During the observation period, 1200 subjects experienced hospitalization at least once, and 134 did not. Table 1 shows the baseline characteristics between the groups. The group that experienced hospitalization was significantly older than the nonhospitalized group (76.8 ± 8.5 years and 73.0 ± 9.8 years, respectively; *P* < .001). The education period of the hospitalized group was significantly shorter than the nonhospitalized group (8.6 ± 5.1 years and 9.7 ± 5.3 years, respectively; *P* = .027). In addition, the hospitalized group showed a significantly higher prevalence of hypertension (79.6% vs. 59.0%; *P* < .001) and diabetes (33.8% vs. 19.4%; *P* = .001). The hospitalized group also showed that the subcortical ischemic burden tended to be more severe (*P* = .017). *APOE* genotype and initial neuropsychiatric evaluation results did not significantly differ between the two groups. There’s no difference in the observation period between hospitalized and nonhospitalized groups (2.3 ± 1.6 years and 2.3 ± 1.7 years, respectively; *P* = .962).

**Table 1:** Baseline characteristics

	Nonhospitalized (*n* = 134)	Hospitalized (*n* = 1200)	*P* value
Age, years	73.0 ± 9.8	76.8 ± 8.5	< 0.001
Male, *n* (%)	42 (31.3)	360 (30.0)	0.824
Education, years (*n* = 134:1185)	9.7 ± 5.3	8.6 ± 5.1	0.027
*APOE*ε4, *n* (%)			0.080
0	84 (62.7)	853 (71.1)	
1	46 (34.3)	304 (25.3)	
2	4 (3.0)	43 (3.6)	
*APOE*ε2, *n* (%)			0.538
0	117 (87.3)	1071 (89.2)	
1	17 (12.7)	124 (10.3)	
2	0 (0.0)	5 (0.4)	
Hypertension, *n* (%)	79 (59.0)	955 (79.6)	< 0.001
Diabetes, *n* (%)	26 (19.4)	405 (33.8)	0.001
Ischemic burden, *n* (%) (*n* = 104:964)			0.017
1	85 (81.7)	658 (68.3)	
2	14 (13.5)	239 (24.8)	
3	5 (4.8)	67 (7.0)	
MMSE (*n* = 133:1171)	23.4 ± 5.9	22.7 ± 5.5	0.175
CDR-SB (*n* = 133:1172)	2.8 ± 3.3	3.1 ± 3.4	0.344
CDR (*n* = 133:1172)	0.7 ± 0.5	0.7 ± 0.6	0.600
KIADL (*n* = 78:614)	0.6 ± 0.7	0.7 ± 0.7	0.305

*Abbreviations:* *APOE* Apolipoprotein E, *CDR* Clinical dementia rating, *CDR-SB* Clinical dementia rating-sum of boxes, *KIADL* Korean-instrumental activities of daily living, *MMSE* Mini-mental state examination

### Hospitalization and cognitive decline

The category of diseases of the circulatory system (ICD-10-CM block: I00–I99) was the most common primary diagnosis of hospitalization (12.73%). The category of diseases of the eye and adnexa (ICD-10-CM block: H00–H59) was next at 12.40%. Then, the category of injury, poisoning, and certain other consequences of external causes (ICD-10-CM block: S00–T88) was the third most common primary diagnosis of hospitalization (11.81%). The entire categories and proportion of primary diagnoses related to all hospitalization in this study are presented in Table S1.

**Fig. 2:**
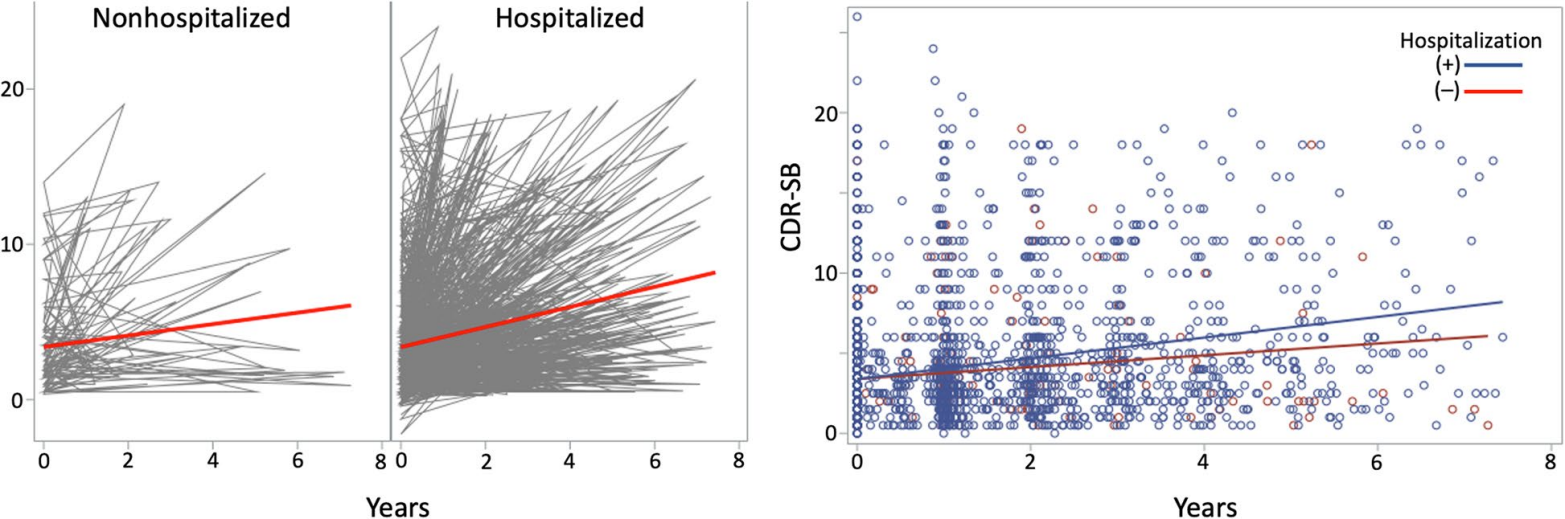
Rate of clinical dementia rating-sum of boxes (CDR-SB) increment. Hospitalized group show a higher increment rate of CDR-SB

The CDR-SB score was significantly increased when the patient experienced a hospitalization (*β* = 1.5083, *P* < .001) (Fig. 2). The same tendencies were shown for the total number of hospitalizations (*β* = 0.0208, *P* < .001) and total hospitalization days (*β* = 0.0022, *P* < .001) (Table 2).

**Table 2:** Mixed models for estimating cognitive changes according to the hospitalization-related variables

Variables of interest	Models	*β*	SE	*P* value
Hospitalization or not	Model I^a^	1.0141	0.0458	< 0.001
	Model II^b^	1.0144	0.0458	< 0.001
	Model III^c^	1.0583	0.0492	< 0.001
Total number of hospitalizations	Model I^a^	0.0246	0.0053	< 0.001
	Model II^b^	0.0246	0.0053	< 0.001
	Model III^c^	0.0208	0.0056	< 0.001
Hospitalization days	Model I^a^	0.0024	0.0004	< 0.001
	Model II^b^	0.0024	0.0004	< 0.001
	Model III^c^	0.0022	0.0004	< 0.001

*Abbreviations:* *APOE* Apolipoprotein E, *CDR-SB* Clinical dementia rating-sum of boxes, *SE* Standard error

^a^Model I: CDR-SB = variable of interest × time + variable of interest + time + sex + CDR-SB (baseline)

^b^Model II: CDR-SB = Model I + *APOE*ε*4* + age + hypertension + diabetes

^c^Model III: CDR-SB = Model II + education year + ischemic burden

Hospitalization showed the same results for CDR grade (*β* = 0.1773, *P* < .001), MMSE score (*β* = − 1.2327, *P* < .001), and KIADL score (*β* = 0.2983, *P* < .001); these neuropsychiatric results more rapidly declined in subjects who experienced hospitalization (Table S2). In the analysis regarding the number of hospitalizations, only the CDR grade (*β* = 0.0045, *P* < .001) showed a significant association with faster cognitive decline, and MMSE and KIADL scores were not statistically significant (Table S3). In the analysis regarding total hospitalization days, a more noticeable cognitive decline was found in CDR grade (*β* = 0.0005, *P* < .001) and MMSE score (*β* = − 0.0013, *P* = .004) than in the KIADL score, which was statistically significant (Table S4).

### Subgroup analysis with hospitalization accompanying delirium or surgery

We performed a subgroup analysis according to the type of hospitalization. The CDR-SB score was significantly higher in all subjects who experienced hospitalization, regardless of whether delirium was present (*β* = 1.5070, *P* < .001) or not (*β* = 1.1028, *P* < .001) compared with the nonhospitalized group. Subjects who experienced hospitalization with delirium showed a faster cognitive decline than subjects who experienced hospitalization without it (Table 3).

**Table 3:** Mixed models for estimating cognitive changes according to whether patients had an experience of hospitalization with or without delirium

Models	Hospitalization without delirium			Hospitalization with delirium		
	*β*	SE	*P* value	*β*	SE	*P* value
Model I^a^	0.9660	0.0480	< .001^d^	1.475	0.1486	< .001^d^
Model II^b^	0.9664	0.0481	< .001^d^	1.476	0.1487	< .001^d^
Model III^c^	1.0128	0.0516	< .001^d^	1.507	0.1612	< .001^d^

*Abbreviations:* *APOE* Apolipoprotein E, *CDR-SB* Clinical dementia rating-sum of boxes, *SE* Standard error

^a^Model I: CDR-SB = hospitalization × time + hospitalization + time + sex + CDR-SB (baseline)

^b^Model II: CDR-SB = Model I + *APOE*ε4 + age + hypertension + diabetes

^c^Model III: CDR-SB = Model II + education year + ischemic burden

^d^Statistically significant difference between hospitalization with and without delirium

Similarly, the CDR-SB score was significantly higher in all subjects who experienced hospitalization, regardless of whether surgery accompanied by general anesthesia was performed (*β* = 0.8634, *P* < .001) or not (*β* = 1.2050, *P* < .001) compared with the nonhospitalized group. Subjects who experienced hospitalization without surgery showed a faster cognitive decline than subjects who experienced hospitalization with surgery (Table 4).

**Table 4:** Mixed models for estimating cognitive changes according to whether patients had an experience of hospitalization with or without surgery

Models	Hospitalization without surgery			Hospitalization with surgery		
	*β*	SE	*P* value	*β*	SE	*P* value
Model I^a^	1.1485	0.0603	< .001^d^	0.8376	0.0691	< .001^d^
Model II^b^	1.1490	0.0604	< .001^d^	0.8378	0.0691	< .001^d^
Model III^c^	1.2050	0.0647	< .001^d^	0.8634	0.0743	< .001^d^

*Abbreviations:* *APOE* Apolipoprotein E, *CDR-SB* Clinical dementia rating-sum of boxes, *SE* Standard error

^a^Model I: CDR-SB = hospitalization × time + hospitalization + time + sex + CDR-SB (baseline)

^b^Model II: CDR-SB = Model I + *APOE*ε4 + age + hypertension + diabetes

^c^Model III: CDR-SB = Model II + education year + ischemic burden

^d^Statistically significant difference between hospitalization with and without surgery

For CDR grade, MMSE score, and KIADL score, hospitalization both with and without delirium was significantly associated with faster cognitive decline than the nonhospitalized group. The CDR grade and KIADL score were significantly aggravated in the hospitalization group with delirium than in the hospitalization group without it (Table S5). For CDR grade, MMSE score, and KIADL score, hospitalization both with and without surgery was significantly associated with faster cognitive decline than the nonhospitalized group. In addition, CDR grade, MMSE score, and KIADL score were all associated with faster-aggravated results in the group hospitalized without surgery compared with the group hospitalized with surgery (Table S6).

## Discussion

In this study, we demonstrated that hospitalization is an exacerbating factor for cognitive decline in older adults. In addition, quantitative indices for hospitalization representing the severity of the physical conditions–number of hospitalizations and total days of hospitalization–were associated with cognitive decline. These results were consistent regardless of whether hospitalization was accompanied by delirium or surgery. Moreover, various cognitive scales also showed generally consistent results.

Several previous studies have attempted to elucidate the relationship between hospitalization and cognitive decline. According to a study by Sprung et al. [18], hospitalization in older adults was found to be related to a decrease in both global and specific domains of cognitive function. Our study also showed similar results with various neuropsychiatric evaluation items such as MMSE score, CDR grade, and KIADL score.

Meanwhile, Richardson et al. [19] reported that hospitalization with delirium significantly accelerated cognitive decline compared with hospitalization without it, consistent with our results. However, in our study, hospitalization without delirium was also related to cognitive decline compared to the nonhospitalized group, a result which is inconsistent with Richardson et al. James et al. [20] argued that a higher hospitalization rate was associated with more rapid cognitive decline. They also reported that nonelective hospitalization was associated with faster cognitive decline than elective hospitalization [21]. Unfortunately, we could not directly compare emergent (nonelective) or elective hospitalization due to the nature of the NHIS database. On the other hand, inconsistent results have been reported on the effect of surgical treatment with general anesthesia on cognitive function [22–24]. The heterogeneous scope of surgery definition, consideration of underlying neurologic conditions or not, and different health-care systems could fuel debate among the authors of many previous studies on this topic [25]. In our results, cognitive decline was less severe in hospitalization with surgical treatment compared with hospitalization without it. We surmise that the many elective hospitalization cases in the surgical treatment group affected our result. In addition, we assume in the elective surgery cases that the anesthesiologist checked the patient’s general condition before surgery, and they received surgical treatment in a medically stable state. In our results, the portion of hospitalization accompanied by diagnoses that caused rapid physical deterioration, such as categories of diseases of the circulatory system or injury, poisoning, and certain other consequences of external causes, were higher than in nonhospitalized patients. In the case of hospitalization without surgery, we suggest that the physical condition worsened more, leading to faster cognitive decline.

In our study, quantitative hospitalization indicators (the number of hospitalizations and total days of hospital stay) were consistently associated with more rapid cognitive decline, suggesting that a large amount of hospitalization was associated with more severe cognitive decline. These results were also generally consistent across the global and specific domains of neurocognitive evaluations.

### Strengths and limitations

As a longitudinal study combining Korea’s NHIS database with hospital data, this study had strengths in that we could obtain information on hospitalization and related diagnostic codes objectively. Hospital data have an advantage in that detailed clinical information is available. On the other hand, the fact that the follow-up data are unreliable, as the patient later visits another hospital, is a critical disadvantage of single-hospital data. The NHIS database can overcome the hospital’s data disadvantages by not only accumulating the patient’s data regardless of the type of hospital, but also being able to confirm other information such as the patient’s insurance eligibility and medical expenses [26]. Consequently, we can considerably improve the database’s utility by combining the hospital and NHIS databases [15]. In addition, our study had the advantage of quantifying and analyzing hospitalization-related variables, especially information on the number of hospitalizations and the total days of hospitalization, not simply whether patients were hospitalized or not. Finally, our study is significant in that it showed consistent results using various cognitive scales.

Although previous studies have investigated the relationship between hospitalization and cognitive decline in older adult groups, it has been challenging to elucidate the precise mechanism of this association [27]. Exposure to sedatives and anesthesia, the burden of medications, hospital environment vulnerability to delirium, stress response, and decreased physical fitness have been suggested as possible underlying mechanisms [28–30]. In addition, a previous study suggested that the more hospitalized, the faster the cognitive decline in older adults, which could be related to neuropathologic features such as more tau angle density and neocortical Lewy body pathologies [20]. This study confirmed that hospitalization exacerbated cognitive decline, but the exact mechanism for this condition could not be identified, which is considered as a limitation. This study was conducted in a retrospective design, and due to the nature of the NHIS database, detailed clinical information relating to individual hospitalizations was lacking. Though we presented the category of primary diagnostic codes claimed upon admission, it was difficult to confirm the findings of laboratory or imaging studies at the time of hospitalization. We defined delirium as a claim code for antipsychotics prescription after hospitalization to persons who had not been prescribed them before; therefore, we could only identify a hyperactive subtype of delirium. In the future, systematic prospective studies using detailed clinical data will be required to clarify which types of hospitalization are more precisely related to cognitive decline. Finally, the sample size between the nonhospitalized and hospitalized groups, quite imbalanced, could affect the statistical significance of the results.

## Conclusion

In conclusion, hospitalization was significantly associated with adverse cognitive trajectories in older adults. In particular, hospitalization with delirium or without surgery more strongly affected cognitive decline. These interactions were consistent regardless of cognitive domains. In older adults, hospitalization is associated with decreased physical fitness, leading to cognitive decline. Therefore, for prevention and management of cognitive dysfunction, it is important to prevent various conditions that lead to hospitalization. To clarify the mechanism and validate these results, further systematic and prospective studies are needed.

## Supplementary Information

**Additional file 1:** **Table S1.** Categories of primary diagnosis related to hospitalization in this study. **Table S2.** Mixed models for estimating cognitive changes according to whether patients had an experience of admission to hospital or not. **Table S3.** Mixed models for estimating cognitive changes according to the total number of hospitalizations. **Table S4.** Mixed models for estimating cognitive changes according to the hospitalization days. **Table S5.** Mixed models for estimating cognitive changes according to whether patients had an experience of hospitalization with or without delirium. **Table S6.** Mixed models for estimating cognitive changes according to whether patients had an experience of hospitalization with or without surgery.

**Additional file 2.** SAS codes for the statistical analyses (mixed models) are provided (SAS® software; https://www.sas.com/).

**Additional file 3.** R codes for the statistical analyses are provided (The R Project for Statistical Computing; https://www.R-project.org/).

## Data Availability

The data from this study are not publicly available due to privacy and ethical restrictions of the Korean National Health Insurance data-sharing system. The data set used in this study can only be accessed by an authorized researcher (JHK).

## Abbreviations

APOE: Apolipoprotein E
CDR: Clinical dementia rating
CDR-SB: Clinical dementia rating-sum of boxes
EMR: Electrical medical records
ICD-10-CM: International classification of disease-10 clinical modification
KIADL: Korean-instrumental activities of daily living
MMSE: Mini-mental state examination (MMSE)
NHIS: National health insurance service
