# Supplementary material for: The effect of all-cause hospitalization on cognitive decline in older adults: a longitudinal study using databases of the National Health Insurance Service and the memory clinics of a self-run hospital
Source: BMC Geriatr. 2023 Feb 1;23:61. doi: 10.1186/s12877-022-03701-4 (PMC9890792; doi:10.1186/s12877-022-03701-4)
Supplement: Supplementary file 1 — Additional file 1: Table S1. Categories of primary diagnosis related to hospitalization in this study. Table S2. Mixed models for estimating cognitive changes according to whether patients had an experience of admission to hospital or not. Table S3. Mixed models for estimating cognitive changes according to the total number of hospitalizations. Table S4. Mixed models for estimating cognitive changes according to the hospitalization days. Table S5. Mixed models for estimating cognitive changes according to whether patients had an experience of hospitalization with or without delirium. Table S6. Mixed models for estimating cognitive changes according to whether patients had an experience of hospitalization with or without surgery. [file 12877_2022_3701_MOESM1_ESM.docx]

**Supplementary Materials**

**Table S1.** Categories of primary diagnosis related to hospitalization in this study.

**Table S2.** Mixed models for estimating cognitive changes according to whether patients had an experience of admission to hospital or not.

**Table S3.** Mixed models for estimating cognitive changes according to the total number of hospitalizations.

**Table S4.** Mixed models for estimating cognitive changes according to the hospitalization days.

**Table S5.** Mixed models for estimating cognitive changes according to whether patients had an experience of hospitalization with or without delirium.

**Table S6.** Mixed models for estimating cognitive changes according to whether patients had an experience of hospitalization with or without surgery.

**Table S1.** Categories of primary diagnosis related to hospitalization in this study.

| **ICD-10-CM block** | **Categories** | ***n* (%)** |
| --- | --- | --- |
| A00–B99 | Certain infections and parasitic diseases | 198 (2.43) |
| C00–D49 | Neoplasms | 728 (8.94) |
| D50–D89 | Diseases of the blood and blood-forming organs and certain disorders involving the immune mechanism | 23 (0.28) |
| E00–E89 | Endocrine, nutritional, and metabolic diseases | 260 (3.19) |
| F01–F99 | Mental, Behavioral and Neurodevelopmental disorders | 647 (7.94) |
| G00–G99 | Diseases of the nervous system | 427 (5.24) |
| H00–H59 | Diseases of the eye and adnexa | 1010 (12.40) |
| H60–H95 | Diseases of the ear and mastoid process | 77 (0.95) |
| I00–I99 | Diseases of the circulatory system | 1037 (12.73) |
| J00–J99 | Diseases of the respiratory system | 435 (5.34) |
| K00–K95 | Diseases of the digestive system | 549 (6.74) |
| L00–L99 | Diseases of the skin and subcutaneous tissue | 64 (0.79) |
| M00–M99 | Diseases of the musculoskeletal system and connective tissue | 946 (11.62) |
| N00–N99 | Diseases of the genitourinary system | 427 (5.24) |
| O00–O9A | Pregnancy, childbirth, and puerperium | 4 (0.05) |
| Q00-Q99 | Congenital malformations, deformations, and chromosomal abnormalities | 1 (0.01) |
| R00–R99 | Symptoms, signs, and abnormal clinical laboratory findings, not elsewhere classified | 287 (3.52) |
| S00–T88 | Injury, poisoning, and certain other consequences of external causes | 962 (11.81) |
| V00–Y99 | External causes of morbidity | 1 (0.01) |
| Z00–Z99 | Factors influencing health status and contact with health services | 81 (0.99) |

Abbreviation: ICD-10-CM, International Classification of Disease-10-Clinical Modification.

**Table S2.** Mixed models for estimating cognitive changes according to whether patients had an experience of admission to hospital or not.

| Models | **CDR** | | |  | **MMSE** | | |  | **KIADL** | | |
| --- | --- | --- | --- | --- | --- | --- | --- | --- | --- | --- | --- |
|  | *β* | SE | *P* value |  | *β* | SE | *P* value |  | *β* | SE | *P* value |
| Model I^a^ | 0.1701 | 0.0092 | < .001 |  | −1.1766 | 0.05525 | < .001 |  | 0.2883 | 0.01804 | < .001 |
| Model II^b^ | 0.1700 | 0.0092 | < .001 |  | −1.1765 | 0.0553 | < .001 |  | 0.2883 | 0.01806 | < .001 |
| Model III^c^ | 0.1773 | 0.0099 | < .001 |  | −1.2327 | 0.05872 | < .001 |  | 0.2983 | 0.01942 | < .001 |

^a^Model I: follow-up score = hospitalization × time + hospitalization + time + sex + baseline score

^b^Model II: follow-up score = Model I + *APOE*ε4 + age + hypertension + diabetes

^c^Model III: follow-up score = Model II + education year + ischemic burden

Abbreviations: *APOE*, apolipoprotein E; CDR, clinical dementia rating; KIADL, Korean-instrumental activities of daily living; MMSE, mini-mental state examination; SE, standard error.

**Table S3.** Mixed models for estimating cognitive changes according to the total number of hospitalizations.

| Models | **CDR** | | |  | **MMSE** | | |  | **KIADL** | | |
| --- | --- | --- | --- | --- | --- | --- | --- | --- | --- | --- | --- |
|  | *β* | SE | *P* value |  | *β* | SE | *P* value |  | *β* | SE | *P* value |
| Model I^a^ | 0.0052 | 0.0010 | < .001 |  | −0.0069 | 0.0063 | .278 |  | 0.0007 | 0.0025 | .766 |
| Model II^b^ | 0.0052 | 0.0010 | < .001 |  | −0.0069 | 0.0063 | .278 |  | 0.0007 | 0.0025 | .773 |
| Model III^c^ | 0.0045 | 0.0011 | < .001 |  | −0.0067 | 0.0066 | .309 |  | 0.0008 | 0.0026 | .770 |

^a^Model I: follow-up score = hospitalization × time + hospitalization + time + sex + baseline score

^b^Model II: follow-up score = Model I + *APOE*ε4 + age + hypertension + diabetes

^c^Model III: follow-up score = Model II + education year + ischemic burden

Abbreviations: *APOE*, apolipoprotein E; CDR, clinical dementia rating; KIADL, Korean-instrumental activities of daily living; MMSE, mini-mental state examination; SE, standard error.

**Table S4.** Mixed models for estimating cognitive changes according to the hospitalization days.

| Models | **CDR** | | |  | **MMSE** | | |  | **KIADL** | | |
| --- | --- | --- | --- | --- | --- | --- | --- | --- | --- | --- | --- |
|  | *β* | SE | *P* value |  | *β* | SE | *P* value |  | *β* | SE | *P* value |
| Model I^a^ | 0.0005 | 0.0001 | < .001 |  | −0.0013 | 0.0004 | .003 |  | 0.0002 | 0.0002 | .197 |
| Model II^b^ | 0.0005 | 0.0001 | < .001 |  | −0.0013 | 0.0004 | .003 |  | 0.0002 | 0.0002 | .201 |
| Model III^c^ | 0.0005 | 0.0001 | < 001 |  | −0.0013 | 0.0005 | .004 |  | 0.0002 | 0.0002 | .198 |

^a^Model I: follow-up score = hospitalization × time + hospitalization + time + sex + baseline score

^b^Model II: follow-up score = Model I + *APOE*ε4 + age + hypertension + diabetes

^c^Model III: follow-up score = Model II + education year + ischemic burden

Abbreviations: *APOE*, apolipoprotein E; CDR, clinical dementia rating; KIADL, Korean-instrumental activities of daily living; MMSE, mini-mental state examination; SE, standard error.

**Table S5.** Mixed models for estimating cognitive changes according to whether patients had an experience of hospitalization with or without delirium.

| Tools | Models | **Hospitalization without delirium** | | |  | **Hospitalization with delirium** | | |
| --- | --- | --- | --- | --- | --- | --- | --- | --- |
|  |  | *β* | SE | *P* value |  | *β* | SE | *P* value |
| CDR | Model I^a^ | 0.1616 | 0.0096 | < .001^d^ |  | 0.2558 | 0.0307 | < .001^d^ |
|  | Model II^b^ | 0.1615 | 0.0096 | < .001^d^ |  | 0.2558 | 0.0307 | < .001^d^ |
|  | Model III^c^ | 0.1684 | 0.0103 | < .001^d^ |  | 0.2709 | 0.0334 | < .001^d^ |
| MMSE | Model I^a^ | −1.1723 | 0.0580 | < .001 |  | −1.2200 | 0.1818 | < .001 |
|  | Model II^b^ | −1.1721 | 0.0581 | < .001 |  | −1.2199 | 0.1819 | < .001 |
|  | Model III^c^ | −1.2221 | 0.0616 | < .001 |  | −1.3409 | 0.1946 | < .001 |
| KIADL | Model I^a^ | 0.2811 | 0.0186 | < .001^d^ |  | 0.4075 | 0.0757 | < .001^d^ |
|  | Model II^b^ | 0.2812 | 0.0186 | < .001^d^ |  | 0.4067 | 0.0757 | < .001^d^ |
|  | Model III^c^ | 0.2899 | 0.0199 | < .001^d^ |  | 0.4530 | 0.0847 | < .001^d^ |

^a^Model I: follow-up score = hospitalization × time + hospitalization + time + sex + baseline score

^b^Model II: follow-up score = Model I + *APOE*ε4 + age + hypertension + diabetes

^c^Model III: follow-up score = Model II + education year + ischemic burden

^d^Statistically significant difference between hospitalization with and without delirium

Abbreviations: *APOE*, apolipoprotein E; CDR, clinical dementia rating; KIADL, Korean-instrumental activities of daily living; MMSE, mini-mental state examination; SE, standard error.

**Table S6.** Mixed models for estimating cognitive changes according to whether patients had an experience of hospitalization with or without surgery.

| Tools | Models | **Hospitalization without surgery** | | |  | **Hospitalization with surgery** | | |
| --- | --- | --- | --- | --- | --- | --- | --- | --- |
|  |  | *β* | SE | *P* value |  | *β* | SE | *P* value |
| CDR | Model I^a^ | 0.2013 | 0.0122 | < .001^d^ |  | 0.1288 | 0.0140 | < .001^d^ |
|  | Model II^b^ | 0.2013 | 0.0122 | < .001^d^ |  | 0.1287 | 0.0140 | < .001^d^ |
|  | Model III^c^ | 0.2102 | 0.0130 | < .001^d^ |  | 0.1330 | 0.0150 | < .001^d^ |
| MMSE | Model I^a^ | −1.2938 | 0.0732 | < .001^d^ |  | −1.0224 | 0.0839 | < .001^d^ |
|  | Model II^b^ | −1.2936 | 0.0733 | < .001^d^ |  | −1.0223 | 0.0839 | < .001^d^ |
|  | Model III^c^ | −1.3684 | 0.0778 | < .001^d^ |  | −1.0542 | 0.0890 | < .001^d^ |
| KIADL | Model I^a^ | 0.2981 | 0.0237 | < .001^d^ |  | 0.2748 | 0.0279 | < .001^d^ |
|  | Model II^b^ | 0.2980 | 0.0237 | < .001^d^ |  | 0.2750 | 0.0279 | < .001^d^ |
|  | Model III^c^ | 0.3058 | 0.0255 | < .001^d^ |  | 0.2879 | 0.0300 | < .001^d^ |

^a^Model I: follow-up score = hospitalization × time + hospitalization + time + sex + baseline score

^b^Model II: follow-up score = Model I + *APOE*ε4 + age + hypertension + diabetes

^c^Model III: follow-up score = Model II + education year + ischemic burden

^d^Statistically significant difference between hospitalization with and without surgery

Abbreviations: *APOE*, apolipoprotein E; CDR, clinical dementia rating; KIADL, Korean-instrumental activities of daily living; MMSE, mini-mental state examination; SE, standard error.
